# Relevance of Zearalenone and its modified forms in bakery products

**DOI:** 10.1007/s12550-023-00493-3

**Published:** 2023-06-15

**Authors:** Franz Pfleger, Christine Schwake-Anduschus

**Affiliations:** 1 Association for Cereal Research e.V., Detmold, Germany; 2 Department of Safety and Quality of Cereals, Max Rubner-Institut, Federal Research Institute of Nutrition and Food, Detmold, Germany

**Keywords:** Mycotoxin, ZEN, Phase I and II metabolites, Modified, Baking, Maize

## Abstract

Zearalenone is a frequently occurring and well-known mycotoxin developed in cereals before and during the harvest period by *Fusarium* spp. mainly in maize and wheat. In addition to the main form, various modified forms (phase I and II metabolites) were detected, in some cases in high amounts. These modified forms can be harmful for human health due to their different toxicity, which can be much higher compared to the parent toxin. In addition, the parent toxin can be cleaved from the phase I and II metabolites during digestion. A risk of correlated and additive adverse effects of the metabolites of ZEN phase I and II in humans and animals is evident. ZEN is considered in many studies on its occurrence in grain-based foods and some studies are dedicated to the behavior of ZEN during food processing. This is not the case for the ZEN phase I and II metabolites, which are only included in a few occurrence reports. Their effects during food processing is also only sporadically addressed in studies to date. In addition to the massive lack of data on the occurrence and behavior of ZEN modified forms, there is also a lack of comprehensive clarification of the toxicity of the numerous different ZEN metabolites detected to date. Finally, studies on the fate during digestion of the relevant ZEN metabolites will be important in the future to further clarify their relevance in processed foods such as bakery products.

## Introduction

Mycotoxins are widespread natural toxins and occur in most grains all over the world. Zearalenone (ZEN), a relevant mycotoxin in maize and co-occurring with deoxynivalenol (DON) in wheat, is produced by several *Fusarium* species (Palumbo et al. 2020). The main ZEN producers are *Fusarium graminearum, Fusarium culmorum, Fusarium cerealis, Fusarium equiseti, Fusarium verticillioides*, and *Fusarium incarnatum* (van Alfen 2014). Mycotoxins such as ZEN cause, for example, reduced grain yield or quality parameters (Schmidt et al. 2016). Optimal conditions for the ZEN production include a moisture content of the raw material above 15% with a relative humidity of 70% or more and the availability of minerals including magnesium, zinc, and cobalt. Other factors such as pH value, optimum temperature of 20–30 °C, and oxygen availability also influence fungal growth (Rogowska et al. 2019) and ZEN release.

Based on the food matrix, some product groups pose a higher risk to unintended ZEN consumption than others. In particular, whole meal cereals may not only be a risk factor for the occurrence of ZEN, but also gluten-free breads made from maize–dough recipes may be a relevant entry source. In addition to the common mycotoxins (e.g., ZEN, DON, and Fumonisins), which are well studied and classified in European guidelines and regulations, there are various modifications that pose difficulties not only in terms of detection and toxicity.

The toxicity of ZEN is primarily attributed to its ability to mimic the effects of the hormone estrogen. Ingested with food, ZEN has the ability to affect the human metabolism and cause adverse effects in several ways. This can lead to a variety of reproductive and developmental problems, particularly in animals. In female animals, ZEN exposure can lead to estrus synchronization, irregular cycles, infertility, and even abortion. In male animals, zearalenone exposure can lead to reduced sperm production and quality (Zhou et al. 2022). ZEN toxicity has also been linked to liver damage, immune system dysfunction, and cancer. Long-term exposure to ZEN has been associated with an increased risk of developing breast cancer in humans (Belhassen et al. 2015).

Apart from the free forms and the matrix-associated mycotoxins, which exist as physically dissolved or trapped complexes, mycotoxins that have undergone biological or chemical modification are referred to as modified mycotoxins (Rychlik et al. 2014). Recently, Palumbo et al. (2020) performed a comparison between published studies on the parent toxin ZEN and its modified forms. Modified forms were considered in less than three papers per year, compared to ZEN with 10 to 20 papers from 2009 to 2015 (Palumbo et al. 2020). Nevertheless, the interest in modified mycotoxins has increased in recent years. This article aims to provide an overview of the current knowledge on phase I and II metabolites of ZEN in general and addresses current issues due to detection, quantification and food processing in bakery products. Due to the lack of reliable information on their occurrence and behavior, the European Food Safety Authority (EFSA) recommends that these compounds should be considered in the safety assessment.

### Structure of phase I and II metabolites of ZEN

Modified forms of ZEN can be divided into phase I and phase II metabolites.

**Fig. 1:**
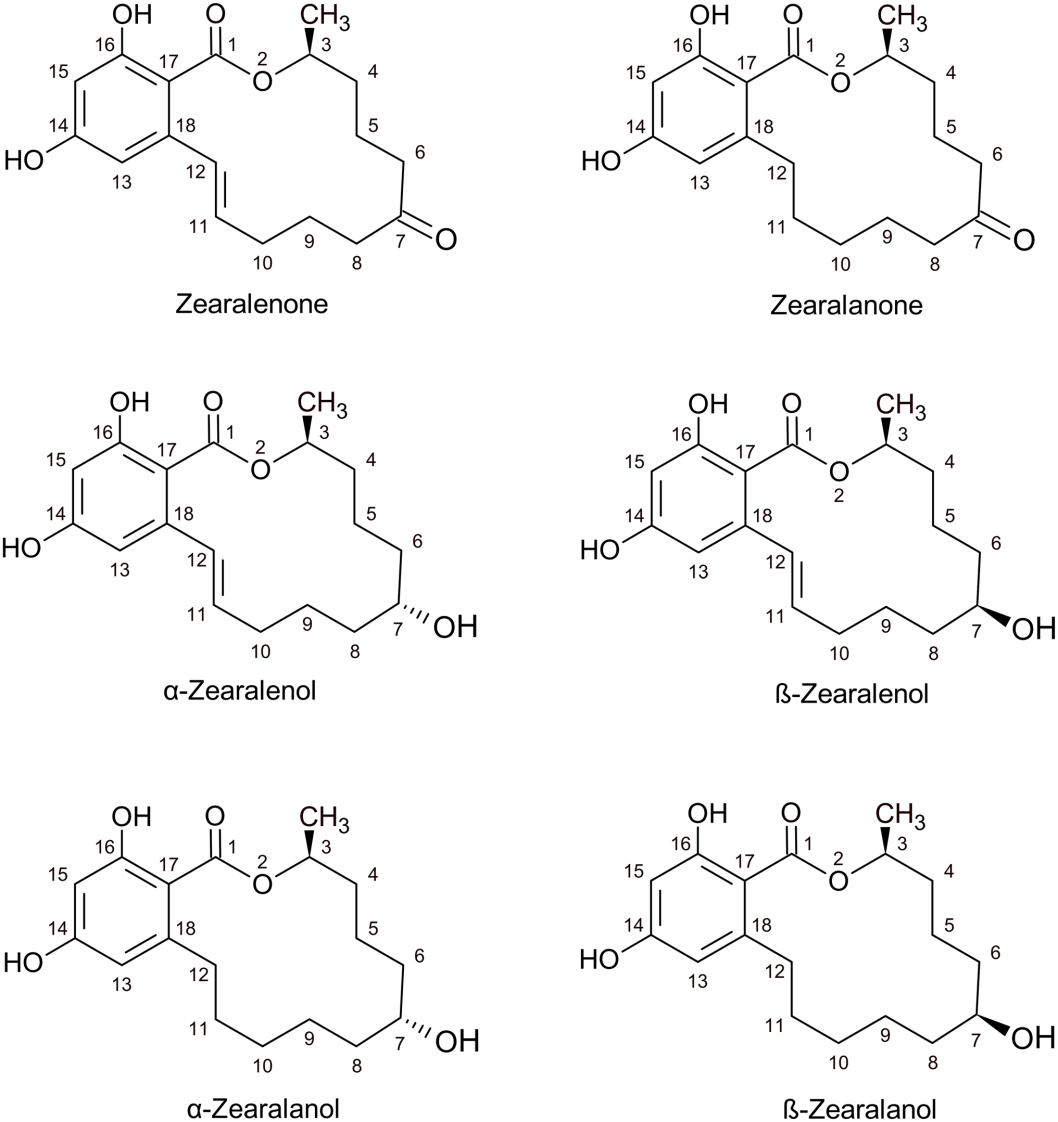
Chemical structure of ZEN and its main phase I metabolites

Phase I metabolites are reduction products of ZEN and may, besides ZEN as parent toxin, serve as initial compounds of phase II metabolism. The most relevant phase I metabolites of the parent toxin ZEN are shown in Fig. 1. The ZEN structure is partly transformed into different patterns.

Other examples of phase I metabolites of ZEN are 5-hydroxy-ZEN, 10-hydroxy-ZEN, ZEN 11,12-oxide, and its hydrolysis products (EFSA 2016). The toxicity of phase I metabolites differs depending on their structure (Berthiller et al. 2013). The differences in toxicity are influenced by the presence and position of functional groups on the zearalenone molecule. In general, the presence of an alpha, beta-unsaturated ketone group is essential for the toxicity of the modified forms. This functional group can react with cellular components and cause damage, leading to toxic effects (Schultz and Yarbrough 2004). These different stereochemical configurations can cause different biological activities and toxicities. For example, the stereochemistry of the modified forms of zearalenone may affect their binding affinity to cellular receptors and alter their toxicity.

**Fig. 2:**
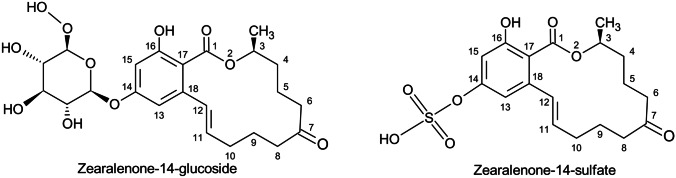
Chemical structure of two ZEN phase II metabolites

The nature and extent of toxicological damage resulting from exposure to ZEN and its modified forms is dependent on the specific type of toxicity exhibited. In terms of reproductive toxicity, it has been established that ZEN and its modified forms can bind to estrogen receptors, thereby inducing estrogenic effects. This can also lead to cytotoxic effects that results in lipid peroxidation (Ahmad et al. 2018; Hou et al. 2013). Furthermore, it has been observed that competitive binding products arising from the interaction between ZEN and estrogen receptors can also bind to the DNA template, modulating the transcription of uterine target genes and protein synthesis (Li et al. 2015). ZEN has been found to bind to estrogen receptors (ERs) located on the surface of immune system cells, resulting in the modulation of various metabolic pathways that contribute to the immune response (Hueza et al. 2014) However, it was stated that especially α-ZEL has a 60 fold higher toxicity than the parent toxin (EFSA 2016). Two conjugated forms of ZEN phase II metabolites are shown exemplary in Fig. 2. Compared to ZEN or the phase I metabolites, these forms are extended by a covalently bound substance such as sulfate or glucoside residues.

Some already known phase II metabolites of ZEN are shown in Table 1. Several of these metabolites need to be structurally defined with further analysis, as they have not yet been fully characterized. In addition, even more glucoside or other carbohydrate molecules can be chemically bound to the conjugates, resulting in more transformed structures. Phase II metabolites, e.g., Z-14-G can accumulate in tissues of the stomach, large intestine and small intestine via transformation to ZEN and/or α-ZEL. The hydrolysis of Z-14-G into ZEN and α-ZEL can lead to a higher systemic ZEN toxicity (Lu et al. 2022). Further results showed that Z-14-G cannot enter cells but exerts cytotoxicity through deglycosylation (Ruan et al. 2022). Preliminary research findings suggest that Z-14-S does not undergo systemic hydrolysis to form ZEN and is instead directly eliminated from the body (Catteuw et al. 2020).

**Table 1:** Several known phase II metabolites of ZEN, their molecular formula and molar mass (Plasencia and Mirocha 1991; EFSA 2016; Borzekowski et al. 2018; Rolli et al. 2018; Kohn and Bunzel 2020)

*Metabolite*	*Molecular formula*	*Molecular weight*	*Comments*
ZEN-14-S	C_18_H_22_O_8_S	398	Characterization completed
α-ZEL-14-S	C_18_H_24_O_8_S	400	
ZEN-14/16-G	C_24_H_32_O_10_	480	Characterization completed
α-ZEL-14-G	C_24_H_34_O_10_	482	Characterization completed
β-ZEL-14-G	C_24_H_34_O_10_	482	Characterization completed
Hydroxy-ZEN-G	C_24_H_32_O_11_	495	
ZEN-Mal-G	C_27_H_34_O_13_	566	Position of mal-G group unknown
α-ZEL-Mal-G	C_27_H_36_O_13_	568	Position of mal-G group unknown
β-ZEL-Mal-G	C_27_H_36_O_13_	568	Position of mal-G group unknown
ZEN-HexPent	C_29_H_40_O_14_	611	
ZEN-G-Xyl	C_29_H_40_O_14_	612	Position of G-Xyl group unknown
α-ZEL-G-Xyl	C_29_H_42_O_14_	614	Position of G-Xyl group unknown
β-ZEL-G-Xyl	C_29_H_42_O_14_	614	Position of G-Xyl group unknown
ZEN-di-G	C_30_H_42_O_15_	642	Position of di-G group unknown
α-ZEL-di-G	C_30_H_44_O_15_	644	Position of di-G group unknown
β-ZEL-di-G	C_30_H_44_O_15_	644	Position of di-G group unknown
ZEN-Mal-di-G	C_33_H_44_O_18_	727	
ZEL-Mal-di-G	C_33_H_46_O_18_	729	
ZEN-tri-G	C_36_H_52_O_20_	803	
β-ZEL-tri-G	C_36_H_54_O_20_	806	Position of tri-G group unknown
ZEN-di-Mal-di-G	C_36_H_46_O_21_	813	
ZEL-di-Mal-di-G	C_36_H_48_O_21_	815	
ZEN-di-Mal-tri-G	C_39_H_54_O_24_	889	
ZEL-di-Mal-tri-G	C_39_H_56_O_23_	891	

*ZEN* zearalenone, *ZEL* zearalenol, *G* glucose, *S* sulfate, *Mal-G* malonyl-glucose, *di-G* di-glucose, *Xyl* xylose, *tri-G* tri-glucose, *HexPent* hexose-pentose

Thus, there is a risk of correlated, additive adverse effects of ZEN phase II metabolites in humans and animals. A further aspect is based on the varying toxicity depending on their chemical structure. Modified mycotoxins do not have estrogenic effects due to the conjugated form and are described in having a reduced toxicity. However, conjugated forms have been shown to be cleaved during intestinal metabolism and liberated ZEL can contribute to estrogenic effects and toxicity. The fate of phase I and II metabolites is not well known (Stadler et al. 2020). Sun et al. investigated the fate of ZEN-14-glucoside (ZEN-14-G) in rats. Results showed that ZEN-14-G rapidly hydrolyses into ZEN in vivo (Sun et al. 2019). A further study also indicates that phase II metabolites like ZEN-14-G can be converted into its parent toxin ZEN in animal and human liver cells (Yang et al. 2018). The systemic toxicity of ZEN is heavily influenced by the co-occurring modified forms. These findings advocate for the development of risk assessment guidelines and regulatory limits of the modified forms of ZEN. The isolated consideration of ZEN in food safety may lead to incorrect conclusions.

#### Analysis and occurrence of ZEN phase I and II metabolites

The analysis of ZEN and its metabolites is usually carried out by HPLC coupled with tandem mass spectrometry (MS/MS). This offers the advantage of supplementing the methods as soon as new standard substances are available.

Different standard methods for ZEN and its modified forms are shown with reference to the food matrix in Table 2.

**Table 2:** A selection of commonly used determination methods for the simultaneous detection of zearalenone and its modified forms

*Analytes*	*Food matrix*	*Extraction mixture*	*Separation technique*	*Detection technique*	*LOD (µg/kg)*	*Reference*
ZEN, ZEN-14-G, ZEN-14-S	Wheat/maize	Acetonitrile/water (75/25)	LC (RP-C18): methanol/5 mM aqueous ammonium acetate (linear gradient)	QTrap-MS/MS	0.1–2	Sulyok et al. (2006)
ZEN, ZEN-14-G,ZEN-14-S	Cereals, cereal-based products	Acetonitrile/water (acetic acid)	LC (RP-C18): methanol/water/acetic acid added with ammonium acetate 10 mM	QTrap-MS/MS	0.5–10	Vendl et al. (2009)
ZEN, ZEN-14-G, ZEN-16-G, ZEN-14-S, α-ZEL-14-G, α-ZEL-14-S, β-ZEL-14-G, β-ZEL-14-S	Cereals, nut products	Acetonitrile/water/acetic acid 79: 20: 1	LC (RP-C18): acetonitrile/ammonium acetate 10 mM	Triple quadrupole ESI source	0.1–2	Liao et al. (2015)
ZEN, ZEN-14-G ZEN-16-G, ZEN-14-S	Wheat flour	Acetonitril/ water 80:20	LC (RP-Phenyl-hexyl) methanol/water and ammonium acetate 5 mM	Triple quadrupole MS/MS	0.2–0.6	Schwake-Anduschus et al. (2015)
ZEN, α-ZEL, β-ZEL, ZEN-14-G, ZEN-14-S, ZEN-16-G, α-ZEL-14-G, β-ZEL-14-G	Barley, oat, wheat	Acetonitrile/water/ acetic acid (79:20:1, v/v/v)	LC (RP-C18) acetonitrile/water buffered with 10 mM ammonium acetate	Triple quadrupole MS/MS	0.3–5	Nathanail et al. (2015)
ZEN, ZEN-14-S, α-ZEL, α-ZEL-14-S, β-ZEL, β-ZEL-14-S	Enzyme-treated oat and wheat based baby food	Acetonitrile/water 50: 50, plus magnesium sulfate	LC (RP-fluoro phenyl) Water/ methanol, both containing 0.1% formic acid and 300 mg/L ammonium formate	QTrap MS/MS	0.3–18	Pascari et al. (2023)

*ZEN* zearalenone, *ZEL* zearalenol, *G* glucose, *S* sulfate

Usually, ZEN and most phase I metabolites can be accurately analysed through routine analysis by comparing with standards. Phase II metabolites place higher demands on analytical technology, mainly because not all standards are available. Identification and determination of not commercially available phase II metabolites usually require the application of further spectroscopic methods, e.g. nuclear magnetic resonance (NMR). In addition, the use of time-of-flight (ToF) detectors can be very helpful in structural elucidation.

ZEN and ZEN-14-sulfate were shown to be located in a significantly higher amount in the fibre-rich fractions (Schwake-Anduschus et al. 2015). Interestingly, in this study, ZEN-sulfate was the only modified form of ZEN detected in the naturally contaminated wheat grains (Schwake-Anduschus et al. 2015). Other studies revealed that the highest contents of ZEN were found in germ, bran and maize as feed compared to lower contents in coarse grit, fine grit, and flour (Brera et al. 2006). These results are important regarding the further use of different maize fractions in food processing. In different food products from two subsequent years the ratio of ZEN‐14‐G to ZEN was highly variable and ranged in 2010 from 1: 36 to 1: 1.3 in 2011 (Binder et al. 2017). In feed raw materials like soybeans, maize and wheat, maximum ZEN-14-sulfate to ZEN ratios were 1: 0.5, 1: 3, and 1: 15, respectively (De Boevre et al. 2012).

### Degradation and reduction of ZEN through cleaning and processing

The pretreatment of grains is an effective factor to minimize ZEN contents even before the processing. Grain cleaning proved to be an effective step to reduce fungal contamination by a factor of 1.2–2 times compared to the grain before cleaning (Scarpino et al. 2021). Treatments like sorting, sieving, and washing could be partly successful to reduce ZEN (Trenholm et al. 1991, 1992). Steeping, milling and heating have proven to be effective methods to decrease the ZEN contents of maize (Karlovsky et al. 2016). These effects depend also on the distribution of ZEN in the maize kernels as a crucial factor for food safety. However, no similar studies for ZEN phase I and II metabolites were performed.

ZEN is described as thermostable in literature, but many food processes such as milling, brewing, cooking, baking, frying, roasting, flaking, alkaline cooking, nixtamalization, and extrusion can affect its content. Heat in combination with the matrix seems to be the most efficient factor. It was shown that baking processes can have enormous impact on the formation, degradation and modification of mycotoxins in grains with a decreasing rate of ZEN in wheat bread of up to 89% during the baking process. Unfortunately, the modified forms of ZEN were not included in this research (Bol et al. 2016).

Therefore, the following processing impacts are related to ZEN due to the lack of data on phase I and II metabolites of ZEN.

The degradation of ZEN in food, i.e., bakery products, depends on several parameters. As mentioned before, heating seems to be a promising influencing factor in reducing ZEN. A number of studies have confirmed a significant decrease in ZEN at high temperatures (Numanoglu et al. 2013; El-Desouky et al. 2012).

Combinations with high pressure, i.e., extrusion processes, could lead to a more effective reduction of ZEN of 56–80% ( Pleadin et al. 2019). Thereby, temperature penetration is an important factor in achieving an effect of the ZEN compounds. Smaller or flatter products tend to have a higher heat transfer, resulting in a stronger ZEN reduction effect of 32% at 200 °C for 30 min compared to 200 °C for 7 min (Numanoglu et al. 2013). This aspect depends on the process time of fermentation and/or baking. In both cases, a decrease in ZEN was observed (Cano-Sancho et al. 2013). Finally, the concentration of ZEN must also be considered. Higher initial concentrations in the food lead to a higher reduction of ZEN (Milani and Maleki 2014).

Several studies have shown the influences of physical and chemical parameters, like lower heat levels, on the ZEN content in foods. Most studies have been carried out in aqueous models, which are difficult to compare with the bread matrices. Ryu et al. described the influence of heat on food matrices by cooking at temperatures of 150 °C and lower. The reduction in ZEN ranged from 0 to 28% (Ryu et al. 1999). Higher degradation rates of 34–68% of ZEN were shown by Smith et al. with temperatures above 150 °C (Smith and Bryant 1975). Even higher temperatures than 175 °C could reduce the ZEN content by more than 92% (El-Desouky et al. 2012). For lower temperatures during processing, further studies at 110 °C for 12 days showed no decreasing effects on the ZEN content. Numanoglu et al. also demonstrated that temperatures of 100 °C did not lead to a degradation of ZEN content (Numanoglu et al. 2013). There is a lack of data on the effects of physical influences on the degradation or formation of ZEN phase I and II metabolites.

The reduction or conversion of ZEN and its modified forms has also been described by using microbiological agents such as yeasts or enzymes. These effects have been demonstrated after the fermentation and in the baking process (Heidari et al. 2014). *Saccharomyces cerevisiae* is able to reduce ZEN levels in bread during fermentation. In this case, longer fermentation times and higher temperatures led to a stronger effect (El-Desouky et al. 2012). Enzymes like ZENG, a ZEN-degrading lactonase named from *G. roseum,* also showed a high degradation performance (Zhang et al. 2020). But the resulting degradation or conversion products have not been characterized, so that no statement can be made about their safety.

### Bread

Different studies report inconsistent results regarding the fermentation of bread and the resulting ZEN content. Cano-Sancho et al. described a total reduction of ZEN at fermentation conditions of 95 min and 25 °C (Cano-Sancho et al. 2013). A further investigation showed a reduction of only 20% after 90 min at 30 °C (Heidari et al. 2014). The baking process was focused in just a few studies. It has been estimated that 60% of ZEN survives in bread after the baking processes. A reduction of 89% ZEN in wheat bread at temperatures of 220 °C was shown (Bol et al. 2016). It is also hypothesized that high temperatures for short durations may effectively eliminate or reduce ZEN contents, although the impact on the levels of modified forms remains unknown (Magan and Olsen 2004).

### Flatbread

Numanoglu et al. carried out investigations on maize bread (dough discs) under isothermal conditions and estimated the ZEN content at different temperatures and the activation energy for these degradation reactions (Numanoglu et al. 2013). They showed that temperatures of 100 °C (approximately the maximum core temperature of bread) had no effect on the ZEN content. Temperatures of 150 °C had a significant effect on the ZEN content and led to degradation processes. Temperatures of 200 °C for 20–30 min showed a reduction of 30/32%, while temperatures of 250 °C showed a reduction of 28% after 15 min (Numanoglu et al. 2013).

### Cookies/biscuits

Several studies have investigated the influence of the baking temperature in bread, cakes and biscuits made from wheat (Bol et al. 2016). For this, baking temperatures of 170 °C, 220 °C, and 270 °C were evaluated. Finally, temperatures of 270 °C showed the highest overall ZEN reduction in bread, cakes. and biscuits (< 82%) (Bol et al. 2016). Other literature describes that 80% of the ZEN remains in the biscuit. On the other hand, Scudamore et al. found no evidence that ZEN levels in wheat-based biscuits are affected by baking at 245 °C for 5 min (Scudamore et al. 2009). No data were found for modified forms such as phase I and II metabolites of ZEN.

### Tortillas

Tortillas are traditional made of maize and mainly consumed in Mexico and Latin America. Abbas et al. (1988) described ZEN reductions of 59–100% at relatively low baking temperatures of 110–120 °C and baking times of 7 or 8 min on each side. Boiling is assumed to be the main reducing factor due to how they get processed. In a study, the maize was boiled in lime water (containing 2% calcium hydroxide) that degrades the lactone in ZEN (Marniemi and Parkki 1975) and therefore reduces ZEN.

### Maize grits/cornflakes

Studies have shown a high reduction during extrusion cooking at temperatures of 120–160 °C of maize grits or maize meal. In a study of Ryu et al. ZEN contents were reduced by 66–83% (Ryu et al. 1999). These results were confirmed by Cetin and Bullerman 2005 (reduction of 66–81%) and Scudamore et al. (2008) (6–54%).

As previously mentioned, the levels of ZEN and its modified forms can be reduced through various processes, although it remains unclear whether the mycotoxin undergoes degradation or conversion. As long as structural elucidation has not been finished, toxicity of degradation products is unknown. Therefore, more studies need to be done. The verification of this can only be achieved through the structural elucidation of the molecule.

### Discussion of processing

The main objective of this review was to summarize the knowledge on ZEN and its modified forms during the processing of bakery products.

ZEN is a well-studied mycotoxin, supported by a large number of investigations. However, the lack of data on modified forms of ZEN is an increasing issue for food safety aspects. There is still a great need for studies in many product groups in the cereal value chain regarding the importance of phase I and II metabolites and their presumed toxic potential to consumers. The modified forms of ZEN, such as Z-14-G, have the potential to enhance the systemic toxicological risk (Lu et al. 2022). The toxicological assessment of Z-14-S appears to differ from that of Z-14-G due to its distinct degradation pathway (Catteuw et al. 2020).

The importance of ZEN and its metabolites depends on the intended use of the grain. The use of whole grain products, i.e. in bakery products or as animal feed, could be a high-risk factor (EFSA 2016). Furthermore, the relevance of maize as one of the main cereals for gluten-free bakery products can be summarized as high. The high occurrence of ZEN and its more toxic modified form such as α-ZEL could be another risk factor for consumers (Birr et al. 2021). It can be concluded that high concentrations of ZEN could have a high impact on human health (Palumbo et al. 2020). It should be noted that the study of Birr et al. did not look at the phase II metabolites of ZEN and therefore it can be assumed that the total amount of ZEN could be higher. The processing of contaminated cereals may be a risk factor for human consumption, too. There is a general lack of research on the behavior of modified forms of ZEN during food processing. To adequately account for the toxicological risk posed by phase I and II metabolites, additional studies are required.

### Physical influences

Pretreatment plays an important role in maize processing. In terms of food safety, all subsequent processing steps can benefit from it. Bread making is usually divided into three steps: kneading, fermentation and baking. In conclusion, it can be inferred that all processing stages may play a significant role in determining the ZEN content.

ZEN levels in baked goods are strongly influenced by the structure and volume of the product. These parameters have an impact on the core temperature and must be taken into account, especially in relation to the baking temperature. This aspect can be illustrated by the following example of maize bread: ZEN has a melting point of 163–165 °C, a temperature that can only be reached in the crust of bread during the baking process. The crumb temperature is much lower, usually below 98 °C. A decreased ZEN content can be expected in the crust, but the crumb is unlikely to be affected. In this case, sampling plays an important role. At high temperatures of 250 °C and above, it must be remembered that food can only be heated to a certain extent to obtain a product that meets consumer expectations in terms of sensory properties.

Other products with a more even temperature rise and distribution (e.g., flat bread or biscuits) might allow more accurate conclusions to be drawn about the heat influence and its reducing potential of ZEN.

### Chemical influences

The use of chemical methods to reduce ZEN in bakery products has limited potential. The pH is an important factor in reducing ZEN levels (Rogowska et al. 2019). Effects are seen at values of 4 and below and at alkaline levels. It should be noted that low values affect the sensory properties of most bakery products. The reduction/raising of pH values to minimize ZEN levels does not seem to be an appropriate method. However, the ammoniation of maize grains could be a possible way to pretreat the crop before processing (Karlovsky et al. 2016).

### Microbiological influences

In addition to the described degradation processes of ZEN, Warth et al. showed that ZEN could be a risk factor for the baby during pregnancy, as ZEN can be converted to the more active α-ZEL in a placental model (Warth et al. 2019). The gut microbiota appears to play an important role in xenobiotic bioactivity in animals and humans. References show a reduced bioavailability of ZEN-14-G and ZEN-16-G compared to ZEN (Binder et al. 2017).

Several studies have investigated formation and cleavage under different conditions. All of the described influences are potentially capable of promoting an unintended increase in the content of ZEN or its modified forms by changing their ratio (Tan et al. 2021).

### Risk assessment

ZEN and its phase I metabolites are known for their estrogenic effects, which have been investigated in many studies demonstrating its toxicity to humans. ZEN is believed to be released from its modified forms. There are multiple factors that need to be considered when looking at the risk of ZEN in the nutrition. The mycotoxin itself can be a risk factor through the consumption of contaminated goods such as cereals or bakery products. Various degradation and modification processes are possible during processing. Besides the degradation of ZEN, phase I and II metabolites are also of interest and it has been shown that they can be cleaved in human microbiota (Dall'Erta et al. 2013). Furthermore, Binder et al. conducted a study demonstrating the complete hydrolysis of ZEN-14-S, ZEN-14-G and ZEN-16-G to ZEN and other unknown substances in the gastrointestinal tract of pigs (Binder et al. 2017). So far, only some of the very many metabolites of ZEN could be included in the assessment. The reason for this might be the small number of studies on the occurrence and behavior during processing and digestion.

Another aspect is the consumption of contaminated animal products that may come from animals that may have been fed with contaminated grain. The potential risks are controversial and vary from negligible (meat) to alarming levels in chicken meat and eggs (Goyarts et al. 2007; Iqbal et al. 2014). Aspects need to be considered when setting limits for gluten-free cereals such as maize for food or feed.

Legal limits for ZEN are set in Europe by Commission Regulation (EC) No 1881/2006. In 2011, the EFSA Panel on Contaminants in the Food Chain established a group health-based TDI of 0.25 µg/kg body weight, including phase I metabolites. A further review of three human studies by the CONTAM Panel in 2016 did not lead to a change in the definition of the TDI. It was also decided that there is no need for an acute reference dose (ARfD) due to its primarily chronic toxicity (EFSA 2016). Phase II metabolites of ZEN are stated to have no estrogenic effects and are not regulated. As recently recommended by Kohn and Bunzel, the inclusion of, e.g., zeralenone malonyl glucosides in future mycotoxin analyses should be considered because of their potential relevance to food and feed safety (Kohn and Bunzel 2020).

However, one question that needs to be asked is whether the legal limits are sufficient. Most limits (like the Commission Regulation (EC) No 1881/2006) are set for the parental toxin ZEN without considering the modified forms of ZEN. This could be a problem in the light of the toxicity of some metabolites (i.e., α-ZEL) and their partly high occurrence. The group-based TDI (0,25 µg/kg bw) of the CONTAM-Panel includes the most known phase I and II metabolites but does not consider their toxicity. Moreover, the digestion of ZEN and its metabolites is not fully known (EFSA 2016). Furthermore, the fate of ZEN and its metabolites during putative breakdown is unknown, requiring structural elucidation of the molecules. Due to this increasing nutritional risk, it must be considered that the co-occurrence of mycotoxins could be a risk factor for additive, reductive, and synergistic effects of these mycotoxins (Gruber-Dorninger et al. 2019; Palumbo et al. 2020).

This review has argued that ZEN metabolites are likely to have adverse effects on human health, comparable to those of the parent toxin. The risk potential for human food is unknown and needs to be investigated through further studies. General knowledge is limited by a lack of data, particularly in relation to studies of modified mycotoxins compared with the parent toxins and its phase I metabolites. Further investigations concluded that the overall uncertainty associated with their assessment is high (EFSA 2016). Up to date, most scientific publications have been carried out with the parent toxin and without considering its modified forms as a collective. Although, analytical capabilities are limited due to problems with the availability of standards and reference materials, there is a great need for methods that are able to detect the relevant modified forms of mycotoxins in general and specially for ZEN (Kovač et al. 2018). The analytical use of ion trap, high-resolution orbitrap, or time-of-flight mass spectrometers could be helpful to measure as yet unknown products or compounds at a later stage (Stadler et al. 2020). This could provide an opportunity to respond as quickly as possible to upcoming regulatory steps and avoid risks to consumer.

Overall, most studies highlight the need to focus on the modified forms of ZEN. Initial evidence suggests that they may behave similarly to the parent toxin ZEN in food, although the metabolism of ZEN, and in particular the phase I and II metabolites, is not well understood. Investigations using dynamic digestion studies could help to gain insight into these processes and determine the toxic risks. Further studies are needed to better assess the health risk for consumers of cereal-based foods, especially for sensitive consumer groups like people with celiac disease who need to switch to gluten-free products, which are often based on maize and may increase exposure.

## Abbreviations

ZEN: Zearalenone
α-ZEL: α-Zearalenol
β-ZEL: β-Zearalenol
α-ZAL: α-Zearalanol
β-ZAL: β-Zearalanol
ZEN-14-G: Zearalenone-14-glycoside
ZEN-16-G: Zearalenone-16-glycoside
ZEN-14-S: Zearalenone-14-sulfate
DON: Deoxynivalenol
